# A new wood-inhabiting mite species of the genus *Dendroseius* Karg, 1965 (Acari, Mesostigmata, Rhodacaridae) from Central Europe (Slovakia)

**DOI:** 10.3897/zookeys.984.57256

**Published:** 2020-11-04

**Authors:** Peter Mašán

**Affiliations:** 1 Institute of Zoology, Slovak Academy of Sciences, Dúbravská cesta 9, 845-06 Bratislava, Slovakia Slovak Academy of Sciences Bratislava Slovakia

**Keywords:** Description, morphology, poplar tree, saproxylic habitat, systematics

## Abstract

A new rhodacarid mite of the genus *Dendroseius* Karg, 1965, *D. reductus***sp. nov.**, was described based on females found in wood detritus and under bark of dead and dying poplar trees in a flood-plain forest in South Slovakia. The new species is unusual among the known congeners in the specifically formed triramous epistome of which the central projection is reduced in length, truncate, and markedly shorter than lateral ones. In other congeneric species, the anterior margin of the epistome possesses three pointed projections of similar size. A dichotomous key for identification of females of the world species classified in the genus *Dendroseius* is provided.

## Introduction

*Dendroseius* was originally described as a subgenus of *Dendrolaelaps* Halbert, 1915 by Karg in 1965, and treated at the generic level by Hirschmann (1974), Lindquist (1975), Evans and Till (1979), Shcherbak (1980), Karg (1993), and other acarologists. The modern concept of *Dendroseius* is largely based on above cited authors who separated the genus from other “*Dendrolaelaps*-like” genera primarily by the following diagnostic character states: (1) dorsal setae j2 with more posterior position, situated between setae j1 and j3, not in a transverse setal row between j1 and z1; (2) gnathosomal groove on deutosternum with seven transverse furrows of which none is reaching beyond the lateral borders of the groove; (3) movable digit of chelicera with three teeth in addition to the apical hook; (4) straight anterior margin of opisthonotal shield; (5) sperm induction system associated with coxae IV.

*Dendroseius* is a small group of rhodacarid mites, currently includes only six known species, namely *D. reticulatus* Sheals, 1956 (= *D. scotarius* Sheals, 1958) distributed mainly in Western Europe (with sporadical findings in North Africa and Central Europe), *D. badenhorsti* (Ryke, 1962) from South Africa, *D. gujarati* Wiśniewski & Hirschmann, 1989 from India, *D. congoensis* Wiśniewski & Hirschmann, 1992 from under bark of a tree imported to Poland from Africa, *D. amoliensis* Faraji, Sakenin-Chelav & Karg, 2006 from Iran, and *D. vulgaris* Ma, Ho & Wang, 2014 from China. Further species initially described under the subgeneric name *Dendroseius*, namely Dendrolaelaps (Dendroseius) fimetarius Karg, 1965 distributed in Central Europe, is now regarded as a member of the genus *Oligodentatus* Shcherbak, 1980 (see Shcherbak 1980).

*Dendroseius* species display a relatively wide spectrum of habitat specialization. Most original descriptions and subsequent reports are based on specimens found in heterogeneous soil detritus (*D. amoliensis*, *D. reticulatus*, *D. vulgaris*), wood substrates (*D. congoensis*, *D. vulgaris*), and manure or cow dung (*D. badenhorsti*, *D. vulgaris*) (Sheals 1956, 1958; Ryke 1962; Wiśniewski and Hirschmann 1992; Karg 1993; Faraji et al. 2006; Ma et al. 2014). Some species may show a phoretic interaction with insects because the deutonymphs of *D. gujarati* was found on an unidentified scarabaeid beetle (Wiśniewski and Hirschmann 1989).

The purpose of this study is to describe a distinct new species of *Dendroseius* from Slovakia contributing thus to knowledge of Rhodacaridae European fauna. Our finding represents also a first record of the genus *Dendroseius* for Slovakia. An introduction of a new key to the identification of the world species based on females is a supplementary aim of this paper.

## Materials and methods

The mites were extracted from decomposing wood detritus by means of a modified Berlese-Tullgren funnel equipped with a 40-Watt bulb, and preserved in ethyl alcohol. Some specimens were collected by wet pincette from under loosen bark. Before identification, the mites were mounted onto permanent microscope slides, using Swan’s chloral hydrate mounting medium. A Leica DM 1000 light microscope equipped with a Leica EC3 digital camera was used to obtain measurements and photos. Measurements were made from slide-mounted specimens. Lengths of idiosoma and shields were measured along their midlines, and widths at their widest point (if not otherwise specified in the description), legs I–IV from coxal base but without the pretarsal ambulacrum. Idiosomal setae were measured from the bases of their insertions to their tips. Measurements are mostly presented as ranges (minimum to maximum). The terminology of dorsal and ventral chaetotaxy follows Lindquist and Evans (1965), and that for leg and gnathosomal setae follows that of Evans (1963a, 1963b).

## Results

### Dendroseius reductus sp. nov.

Taxon classificationAnimaliaMesostigmataDigamasellidae

404FB3FB-0AFB-5A3F-AF42-FB415EBB4260

http://zoobank.org/8C144F00-0FF6-4D7C-84AB-11A865AE85E1

Figs 1–2 , 3–6 , 7–10

#### Type material examined.

***Holotype*** female: SW Slovakia, Podunajská Rovina Flatland, Bratislava Capital, Rusovce Settlement, hard-wood flood-plain forest (*Fraxino-Ulmetum carpinetosum*) with poplar (*Populus* sp.), 135 m a.s.l., March 7, 2020, detritus from a hollow of old and dying poplar tree. ***Paratype*** females: one specimen, with the same data as for holotype; three specimens, the same locality as in holotype, May 19, 2004, under bark of dead poplar tree. The type material is deposited at the Institute of Zoology, Slovak Academy of Sciences, Bratislava, Slovakia.

#### Description

**Figures 1, 2.**
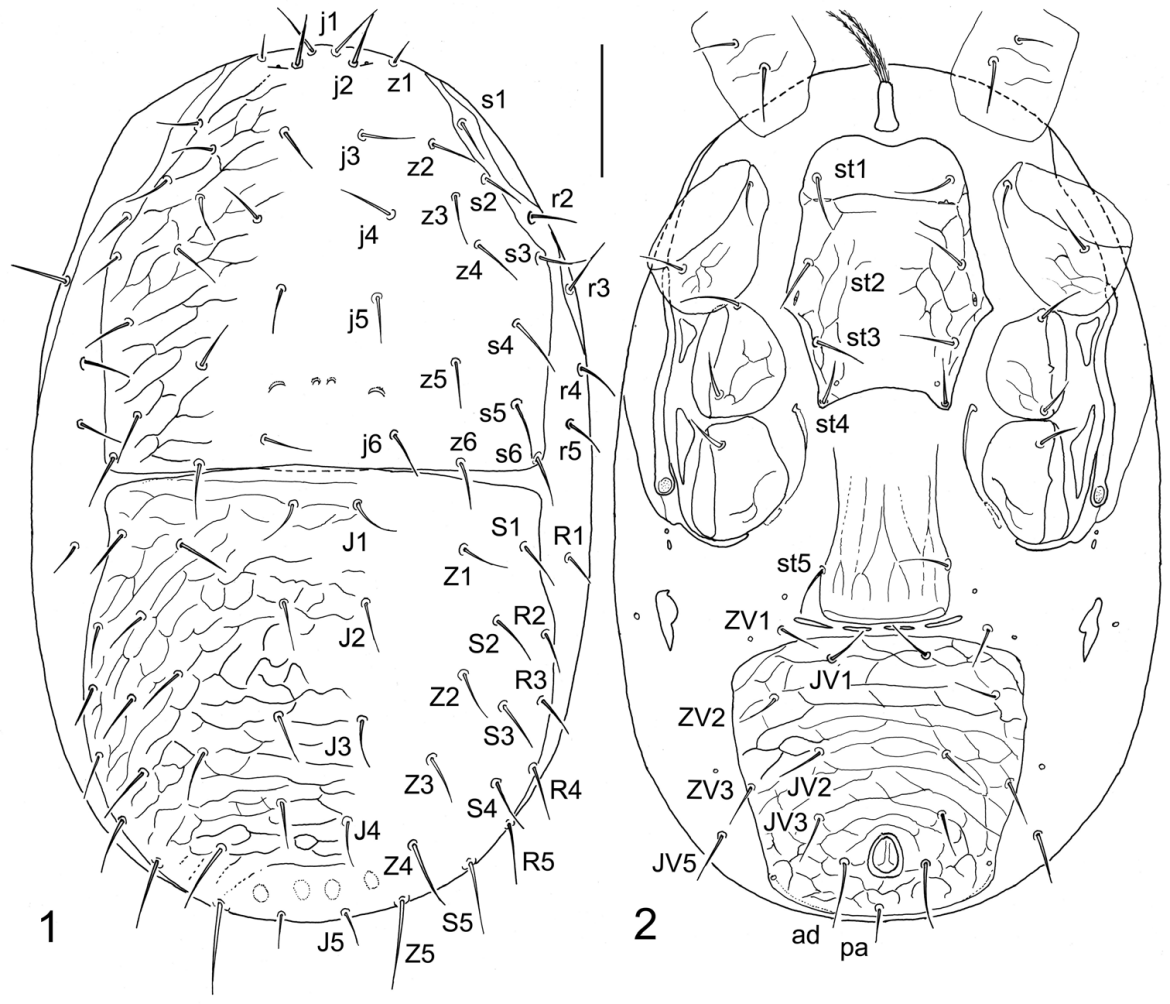
*Dendroseius reductus* sp. nov., female, with setal notation of idiosomal setae **1** dorsal idiosoma **2** ventral idiosoma. Scale bar: 50 µm.

(Female). *Dorsal idiosoma* (Figs 1, 7). Idiosoma oblong, regularly oval, widest at medial portion, 315–345 μm long and 195–220 μm wide (*N* = 5). Dorsal shield completely divided to podonotal and opisthonotal parts, not completely covering dorsal surface, exposing narrow strips of lateral soft integument. Podonotal shield 157–170 μm long and 170–175 μm wide, with smooth medial surface, delicate lateral reticulation, 17–18 pairs of setae (j1–j6, z1–z6, s2–s6, s1 symmetrically or asymmetrically situated on the shield and soft integument, respectively), and two pairs of scleronoduli between setae j5 and j6; outer scleronoduli larger and more conspicuous. Marginal setae r2, r4, r5 and R1 inserted in lateral soft integument, apparently outside the dorsal shields, and humeral setae r3 placed on peritrematal shields. Opisthonotal shield 165–180 μm long and 175–190 μm wide, finely reticulate on whole surface, and bearing 19 pairs of setae (J1–J5, Z1–Z5, S1–S5, R2–R5). All dorsal setae smooth and needle-shaped, mostly similar in their lengths; setae z1 shortest (10–11 μm) and Z5 longest (35–40 μm); the lengths of some selected dorsal setae as follows: j1 16–19 μm, j3 21–23 μm, j5 17–21 μm, r5 24–28 μm, J1–J4 and Z1–Z3 18–23 μm, J5 17–19 μm, Z4 25–29 μm, S1–S4 21–25 μm, S5 27–30 μm, R2–R4 18–20 μm.

**Figures 3–6.**
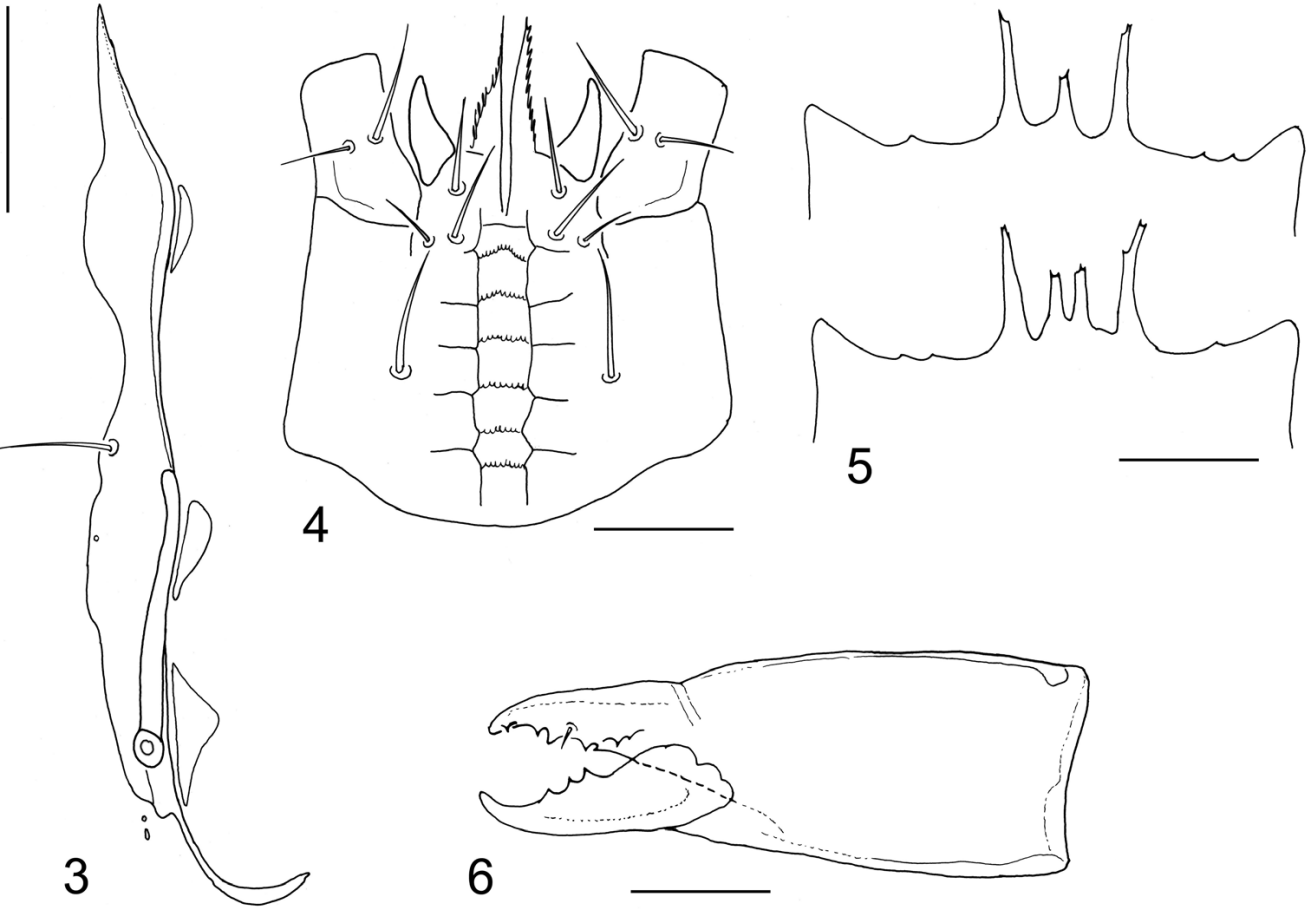
*Dendroseius reductus* sp. nov., female **3** peritrematal shield and adjacent exopodal platelets **4** ventral gnathosoma **5** epistomes, normal form and an aberration having two central prongs **6** chelicera, lateral view. Scale bars: 50 µm (**3**), 25 µm (**4**), 20 µm (**5, 6**).

*Ventral idiosoma* (Figs 2, 9). Tritosternum with long columnar base and two long and distinctly pilose laciniae. Presternal area lacking separate scutal elements. Sternal shield oblong, 90–100 μm long, 66–73 μm wide at level of constriction between coxae II, with smooth and weakly sclerotized but well-defined anteriormost portion possessing first pair of sternal setae and reaching level of first pair of lyrifissures (iv1); posterior margin regularly convex and produced to relatively acute angles each bearing a metasternal seta (st4); the shield with fine reticulate pattern on lateral parts, four pairs of sternal setae (st1–st4) and three pairs of lyrifissures, iv1–iv3 (opening of iv1 and iv2 slit-like while iv3 suboval, iv1 with transverse position to the body axis while iv2 oriented longitudinally). Epigynal shield oblong, 50–60 μm wide, hyaline anteriorly (anterior margin obscure and not distinguishable), almost straight or widely rounded posteriorly, bearing one pair of setae (st5) and a pattern of longitudinal lines; genital lyrifissures (iv5) situated on soft integument behind st5, outside the shield. Four slit-like postgenital sclerites close to posterior margin of epigynal shield present. Peritremes shortened, 66–80 μm long, with anterior end reaching slightly beyond posterior margin of coxa II; peritrematal shields well-developed, free from podonotal shield, markedly narrowed behind coxae IV, bearing humeral setae (r3), and adjacent to anterior margin of podonotal shield close to paravertical setae z1 (Fig. 3). Three subtriangular exopodal platelets between peritrematal shields and coxae present. Metapodal soft integument with a pair of small irregular platelets having longitudinal position. Ventrianal shield subquadrate, only slightly wider than long (105–115 μm long and 112–130 μm wide), delicately reticulate on whole surface, bearing five pairs of pre-anal setae (JV1–JV3, ZV2, ZV3) in addition to three circum-anal setae, and a pair of marginal gland pores (gv3) more or less aligned with posterior margin of anal opening; adanal setae (ad) apparently longer than postanal seta (ad 27–30 μm, pa 15–17 μm). Soft opisthogastrict integument bearing two pairs of setae (ZV1, JV5). Ventrally situated setae similarly formed as those on dorsal side of idiosoma. The lengths of some selected setae on ventrum as follows: st1 25–28 μm, st2 24–27 μm, st3 22–26 μm, st4 21–24 μm, st5 21–23 μm, JV1 and JV2 18–23 μm, JV5 22–27 μm.

*Sperm induction system* (Fig. 10). Each gonoporus associated with inner posterior margin of coxa IV, together with relatively short and broad duct formed as a club-shaped structure; the duct opening into small hyaline sacculus. Sperm system of both coxae mutually connected with membranous structure (Fig. 10).

*Gnathosomal structures* (Figs 4–6, 8). Deutosternal groove with seven transverse sculptural furrows, six posterior ones with tiny denticles; corniculi horn-like, divergent; internal malae with median projections contiguous and with serrate margins (Fig. 4). The lengths of hypostomal setae as follows: h1 17–22 μm, h2 11–14 μm, h3 19–22 μm, pc 21–25 μm; the setae smooth and needle-like. Palp apotele 2-tined. Epistome triramous, with short central and longer lateral branches, each terminally with one to three points; one specimen abnormally with two central branches (Figs 5, 8). Cheliceral digits of similar size, dentate; movable digit with three closely set teeth in addition to distal hook; fixed digit with about seven teeth in addition to apical hook and minute setiform *pilus dentilis* (Fig. 6); a coronet-like fringe, dorsal cheliceral seta and antiaxial lyrifissure not discerned.

**Figures 7–10.**
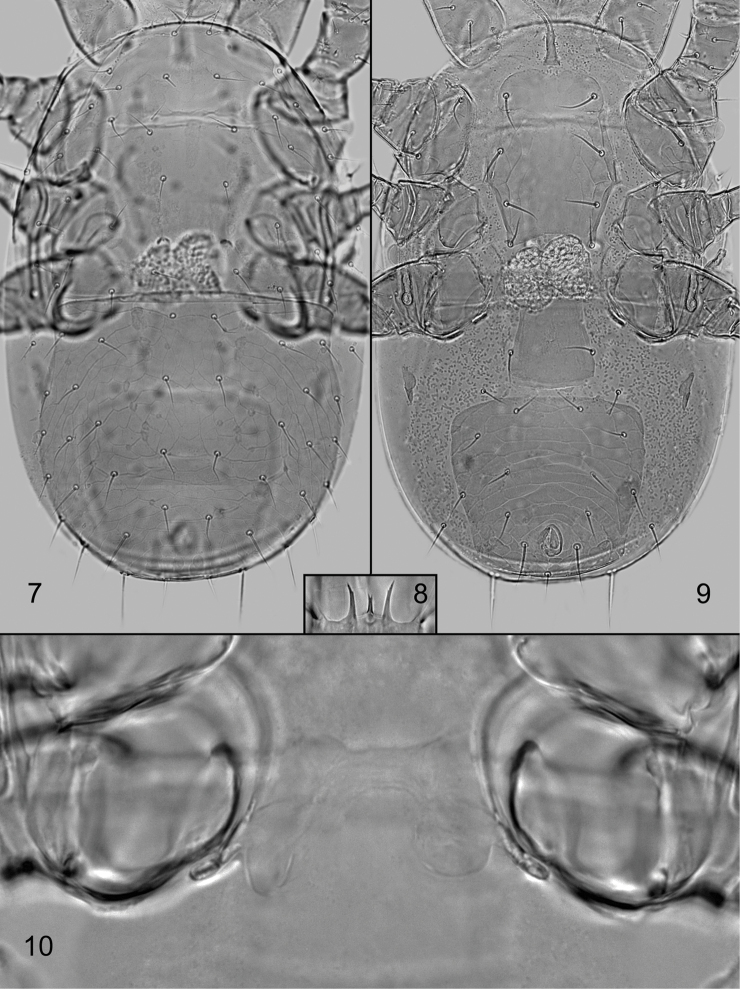
*Dendroseius reductus* sp. nov., photographs of female **7** dorsal idiosoma **8** epistome **9** ventral idiosoma **10** sperm induction system. Not to scale.

*Legs*. All legs with well-developed pretarsus and ambulacral apparatus (including pulvillus and two claws), shorter than idiosoma: legs I 290–310 μm, legs II 210–230 μm, legs III 180–200 μm, and legs IV 260–285 μm long. Leg segments not spurred ventrally, with normal chaetotactic pattern for the genus: leg I – coxa 0-0/1, 0/1-0 (2), trochanter 1-1/1, 0/2-1 (6), femur 2-3/2, 2/2-2 (13), genu 2-3/2, 2/1-2 (12), tibia 2-3/2, 2/1-2 (12); leg II – coxa 0-0/1, 0/1-0 (2), trochanter 1-0/1, 0/2-1 (5), femur 2-3/1, 2/2-1 (11), genu 2-3/1, 2/1-2 (11), tibia 2-2/1, 2/1-2 (10); leg III – coxa 0-0/1, 0/1-0 (2), trochanter 1-1/1, 0/2-0 (5), femur 1-2/1, 1/0-1 (6), genu 2-2/1, 2/1-1 (9), tibia 2-1/1, 2/1-1 (8); leg IV – coxa 0-0/1, 0/0-0 (1), trochanter 1-1/1, 0/2-0 (5), femur 1-2/1, 1/0-1 (6), genu 1-2/1, 2/0-1 (7), tibia 1-1/1, 2/1-1 (7); tarsi II–IV – 18 setae each. Leg setae uniform and similar in length, smooth and needle-like.

#### Etymology.

The specific name is derived from the Latin word *reductus* (reduced) and expresses an important feature of the species ‒ an unusual shape of epistome, a fine flat structure situated on upper surface of gnathosoma, with partly reduced central projection on its anterior margin.

#### Taxonomic notes.

The triramous epistome of the new species, with remarkably shortened central projection, is unique and quite unlike any other known species in the genus *Dendroseius*. In other congeners, this central projection is much longer and more acuminate in the terminal part, reaching to (in *D. amoliensis*) or slightly beyond the level of the adjacent lateral apices (in all other congeners, including two species exclusively based on deutonymphs and not included in the key below). Nevertheless, the new species is most similar to *D. vulgaris* distributed in China (Ma, Ho and Wang 2014), and it can be distinguished from *D. vulgaris* and other species by the character states presented in the identification key below. Some metric data for *D. reticulatus* provided in the key are derived from specimens in author’s personal collection from Wales, UK (Anglesey, Newborough Beach, found in decomposing plant substrate in a sandy coastal area). The morphological data used for other *Dendroseius* species were based only on the original descriptions.

### Key to world species of *Dendroseius* (females)

**Table:** 

1	Ventrianal shield subtriangular in shape, with posterior margin convex, and four pairs of pre-anal setae (JV1–JV3, ZV2; ZV3 situated outside the shield); peritremes relatively shorter, reaching about the middle of coxae III; length of idiosoma: 367 µm [Iran]	***Dendroseius amoliensis* Faraji, Sakenin-Chelav & Karg, 2006**
−	Ventrianal shield subquadrate or subrectangular in shape, with posterior margin only moderately curved, and five pairs of pre-anal setae (JV1–JV3, ZV2, ZV3); peritremes relatively longer, reaching coxae II	**2**
2	Podonotal soft integument with at most two pairs of setae (r4, r5; r2 situated on dorsal shield); length of idiosoma: 364 µm [South Africa]	***Dendroseius badenhorsti* (Ryke, 1962)**
−	Podonotal soft integument with at least three pairs of setae (r2, r4, r5; sometimes s1)	**3**
3	Dorsal shield setae shorter (J1–J4 normally less than 15 µm in length); setae r5 and Z5 similar in length (26–32 µm); ventrianal shield wider than long (L 95–100 µm; W 120–135 µm), dish-shaped; smaller species, length of idiosoma: 260–290 µm [Europe, North Africa]	***Dendroseius reticulatus* (Sheals, 1956)**
−	Dorsal shield setae longer (J1–J4 normally more than 18 µm in length); setae r5 1.5–2 times shorter than Z5 (r5 23–28 µm, Z5 35–55 µm); ventrianal shield similar in width and length (L 105–143 µm; W 112–135 µm), cup-shaped; larger species, length of idiosoma: 315–375 µm	**4**
4	Central process of epistome shortened, about two times shorter than those on lateral margins; dorsal shield setae generally shorter: J4≈1/2×J4–J5, S1≈1/2×S1–R2, S2≈1/2×S2–S3, S3≈1/2×S3–S4 (j5 17–20 µm, J1–J4 19–23 µm, J5 17–19 µm, Z5 35–40 µm); ventrianal shield slightly wider than long (L 105–115 µm; W 112–130 µm); length of idiosoma: 315–345 µm [Slovakia]	***Dendroseius reductus* sp. nov.**
−	Epistome with three well-developed prongs, central process slightly longer than lateral ones; dorsal shield setae generally longer: J4≈J4–J5, S1≈S1–R2, S2≈S2–S3, S3≈S3–S4 (j5 25–30 µm, J1–J4 23–42 µm, J5 26–38 µm, Z5 48–55 µm); ventrianal shield slightly longer than wide (L 131–143 µm; W 128–135 µm); length of idiosoma: 353–375 µm [Taiwan]	***Dendroseius vulgaris* Ma, Ho & Wang, 2014**

## Supplementary Material

XML Treatment for Dendroseius reductus
